# The pathogenesis linked to coenzyme Q10 insufficiency in iPSC-derived neurons from patients with multiple-system atrophy

**DOI:** 10.1038/s41598-018-32573-1

**Published:** 2018-09-21

**Authors:** Fumiko Kusunoki Nakamoto, Satoshi Okamoto, Jun Mitsui, Takefumi Sone, Mitsuru Ishikawa, Yorihiro Yamamoto, Yumi Kanegae, Yuhki Nakatake, Kent Imaizumi, Hiroyuki Ishiura, Shoji Tsuji, Hideyuki Okano

**Affiliations:** 1 Department of Neurology, University of Tokyo, School of Medicine, Hongo, Bunkyo-ku, Tokyo, 113-8655 Japan; 2 Department of Physiology, Keio University School of Medicine, Shinanomachi, Shinjuku-ku, Tokyo, 160-8582 Japan; 3 School of Bioscience and Biotechnology, Tokyo University of Technology, Katakuramachi, Hachioji City, Tokyo 192-0914 Japan; 4 Research Center for Medical Science, Jikei University School of Medicine, Nishi-shinbashi, Minato-ku, Tokyo, 105-8461 Japan; 5 Department of Systems Medicine, Keio University School of Medicine, Shinanomachi, Shinjuku-ku, Tokyo, 160-8582 Japan

## Abstract

Multiple-system atrophy (MSA) is a neurodegenerative disease characterized by autonomic failure with various combinations of parkinsonism, cerebellar ataxia, and pyramidal dysfunction. We previously reported that functionally impaired variants of *COQ2*, which encodes an essential enzyme in the biosynthetic pathway of coenzyme Q10, are associated with MSA. Here, we report functional deficiencies in mitochondrial respiration and the antioxidative system in induced pluripotent stem cell (iPSC)-derived neurons from an MSA patient with compound heterozygous *COQ2* mutations. The functional deficiencies were rescued by site-specific CRISPR/Cas9-mediated gene corrections. We also report an increase in apoptosis of iPSC-derived neurons from MSA patients. Coenzyme Q10 reduced apoptosis of neurons from the MSA patient with compound heterozygous *COQ2* mutations. Our results reveal that cellular dysfunctions attributable to decreased coenzyme Q10 levels are related to neuronal death in MSA, particularly in patients with *COQ2* variants, and may contribute to the development of therapy using coenzyme Q10 supplementation.

## Introduction

Multiple-system atrophy (MSA) is a neurodegenerative disease characterized by autonomic failure in addition to various combinations of parkinsonism, cerebellar ataxia, and pyramidal dysfunction. The disorder is classified into two subtypes: MSA-C, characterized predominantly by cerebellar ataxia, and MSA-P, characterized predominantly by parkinsonism. The cascade of events that underlie the pathogenesis of MSA remains largely unknown. MSA is characterized by the development of cytoplasmic aggregates of α-synuclein (glial cytoplasmic inclusions; GCIs), primarily in oligodendrocytes^1–3^. It is suggested that α-synuclein plays an important role in the pathogenic cascade of MSA. Moreover, striatum and midbrain neurons are preferentially affected in MSA patients and demonstrate expression of apoptosis-related proteins, indicating induction of apoptosis in neurons of MSA patients^4^.

Although MSA has been defined as a non-genetic disorder until recently, several multiplex families with the disease have been described^5–7^. Moreover, MSA-C has been reported to be more prevalent than MSA-P in the Japanese population, whereas MSA-P has been reported to be more prevalent than MSA-C in Europe and North America^8–11^. These reports indicate that genetic factors confer susceptibility to the disease.

In our previous study, we identified a homozygous mutation (p.[M128V–V393A]/[M128V–V393A]) and compound heterozygous mutations (p.[R387*]/[V393A]) in *COQ2* in two multiplex families^12^. Furthermore, we observed that functionally impaired heterozygous *COQ2* variants were associated with sporadic MSA. Several studies exploring whether the same polymorphism is associated with MSA have been reported^13–19^. Meta-analysis of previous four studies provided evidence that *COQ2* p.V393A is a polymorphism commonly present in East Asian populations in Japan, Taiwan and China and that *COQ2* p.V393A is significantly associated with an increased risk of MSA in East Asian populations^15^.

**Figure 1:**
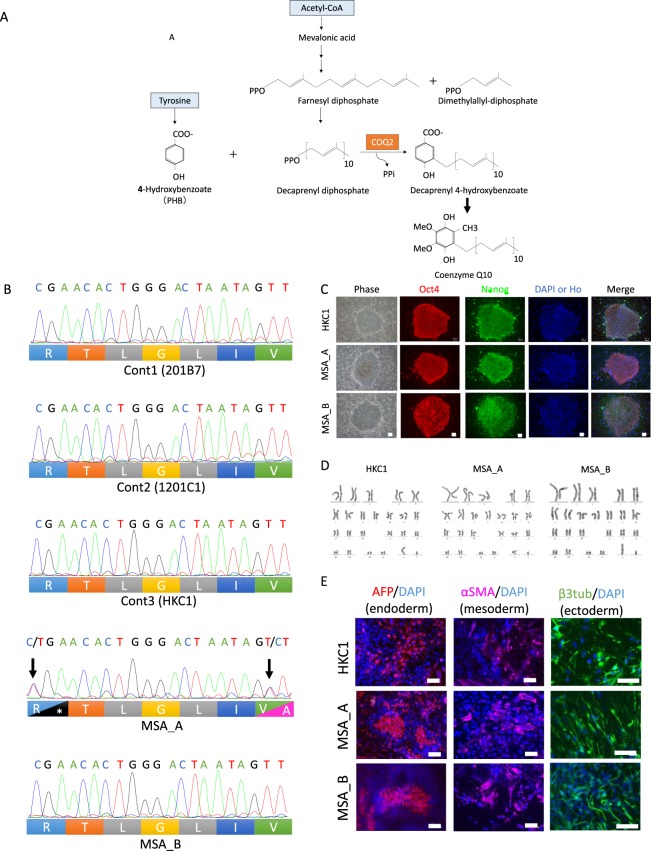
Establishment and characterization of iPSCs from MSA patients and a healthy subject. (**A**) Coenzyme Q10 biosynthesis pathway. Coenzyme Q10 is composed of a benzoquinone and a decaprenyl side chain. COQ2, para-hydroxybenzoate (PHB)-polyprenyl transferase, catalyzes the condensation reaction of PHB and decaprenyl diphosphate. (**B**) Sequencing analysis of genomic DNA from iPSCs of each clone. MSA_A iPSCs harbored heterozygous mutations, c.1159C > T (p.R387*) and c.1178T > C (p.V393A), in exon 7 of the *COQ2* gene (arrows). Three control lines and MSA_B lines did not have mutation in the *COQ2* gene. (**C**) Representative morphology of iPSC colonies and immunochemical analysis of pluripotent stem cell markers, NANOG and OCT4. The scale bars represent 100 µm. Ho, Hoechst33258. (**D**) Karyotypes by the G-band staining method. Each clone shows a normal karyotype. (**E**) The lines showed tridermic markers (endoderm: AFP-positive cells, mesoderm: αSMA-positive cells, ectoderm: βIII-tubulin-positive cells). The scale bars represent 100 µm (endoderm and mesoderm) and 50 µm (ectoderm). β3tub, βIII-tubulin.

The *COQ2* gene encodes the enzyme COQ2 which is essential for the biosynthesis of coenzyme Q10 (Fig. 1A). Furthermore, the activity of COQ2 in lymphoblastoid cell lines with the *COQ2* variant p.V393A, established from patients with MSA, was significantly lower than that in control cell lines, and the level of coenzyme Q10 activity in frozen cerebellum samples obtained from a patient carrying a homozygous mutation was substantially lower than the levels observed in controls without the mutation^12^. Coenzyme Q10 plays an essential role in mitochondrial electron transport and antioxidant actions. Our findings suggest that impaired COQ2 activity, which is predicted to impair the mitochondrial respiratory chain and increase vulnerability to oxidative stress, confers susceptibility to MSA.

Furthermore, recent reports detected decrease levels of coenzyme Q10 in the cerebellum of MSA patients compared to those in controls or other neurodegenerative diseases^20,21^. Our recent data demonstrated decreased levels of plasma coenzyme Q10 in patients with MSA regardless of the *COQ2* genotype^22^, in alignment with a report from another research group that also demonstrated decreased levels of serum coenzyme Q10 in patients with MSA^23^. Besides, a recent study reported reduced coenzyme Q10 levels in cerebrospinal fluid of sporadic MSA patients^24^. These results indicate that decreased levels of coenzyme Q10 are a fundamental characteristic of MSA patients with or without *COQ2* mutations. However, the mechanisms underlying decreased coenzyme Q10 levels in individuals not carrying *COQ2* mutations remain to be elucidated. Thus, it is important to examine cellular functions associated with coenzyme Q10, and the relationship between the pathological mechanisms involved in MSA and the cellular functions of coenzyme Q10 in neural cells of both MSA patients carrying *COQ2* mutations and those without *COQ2* mutations.

To reveal the relationship between the pathologic mechanisms involved in MSA and cellular functions associated with coenzyme Q10, disease-specific induced pluripotent stem cells (iPSCs) were used. Because most patients with MSA do not carry any *COQ2* mutations, genetic factors other than *COQ2* mutations should also be considered. In addition, it is possible to reproduce phenomena that occur in MSA patients *in vivo* and to evaluate the phenomena and the cellular functions associated with coenzyme Q10 simultaneously by using neural cells derived from iPSCs generated from the patients.

In the present study, we generated iPSCs from an MSA patient carrying compound heterozygous *COQ2* mutations (p.[R387*]/[V393A]), an MSA patient without *COQ2* mutations and a healthy volunteer. These iPSCs were differentiated into neural cells *in vitro*, and we analysed coenzyme Q10 levels, cellular functions associated with coenzyme Q10 and apoptosis in neural cells derived from these iPSCs. We also considered the causal relationship between the observed cellular dysfunctions and *COQ2* mutation using the Clustered, Regularly Interspaced, Short Palindromic Repeats (CRISPR)/CRISPR-associated protein 9 (Cas9) system.

## Results

### Clinical features of MSA patients

We established iPSCs from two patients (MSA_A and MSA_B) who were clinically diagnosed with cerebellar type of MSA. MSA_A, an affected member of a previously described Japanese multiplex family with MSA^12,25^, was 61-year-old male. He was observed to have staggering gait, dysarthria, hypotension and erectile dysfunction at the age of 44 years. At the age of 50 years, he was unable to walk. At the age of 56 years, he showed bilateral elbow rigidity and resting tremor of left arm. At the age of 59 years, he showed mild renal dysfunction. He carried compound heterozygous nonsense (p.R387*, c.1159C > T) and missense (p.V393A, c.1178T > C) mutations in *COQ2*. His elder sister who was affected carried the same compound heterozygous p.[V393A]/[R387*] variants of *COQ2*.

MSA_B was a 56-year-old male. He showed staggering gait at the age of 54 years. Dysarthria and coordination disorder were evident at 55 years of age. He also showed limb and truncal ataxia and orthostatic hypotension. MRI of the patient’s brain revealed brain stem atrophy and cross sign of the pons. Sequencing analysis of genomic DNA from the iPSCs derived from the patient confirmed that he did not carry any *COQ2* mutations.

### Generation of iPSCs from MSA patients and CIRSPR/Cas9-mediated correction of *COQ2* mutation

We generated MSA iPSCs from peripheral blood mononuclear cells (PBMCs) isolated from two MSA patients (MSA_A and MSA_B, Table 1). To reprogram the PBMCs into MSA_A-iPSCs and MSA_B-iPSCs, we used episomal vectors carrying *OCT4*, *SOX2*, *KLF4*, *L-MYC*, *LIN28*, and *p53* shRNA. MSA_A had two heterozygous mutations (c.1159C > T and c.1178T > C) in the exon 7 coding sequence of the *COQ2* gene on each allele We confirmed that MSA_A-iPSCs (MSA_A26, MSA_A31, and MSA_A34) harboured compound heterozygous mutations in the *COQ2* gene (Fig. 1B) and had no other mutations in the *COQ2* gene. Furthermore, we established a control iPSC line, in addition to the two control human iPSC lines, 201B7 and 1201C1, which had been established previously^26,27^ (Table 1). These three control lines were derived from three healthy individuals. These three control lines and the MSA_B lines (MSA_B1, MSA_B2, and MSA_B3) did not carry mutations in the *COQ2* gene (Fig. 1B). Importantly, these iPSC lines had properties similar to those of human embryonic stem cell lines, as demonstrated by the expression of pluripotent stem cell markers (OCT4 and NANOG) detected using immunocytochemical analysis (Fig. 1C). Normal karyotypes were confirmed by the G-band staining method (Fig. 1D). We also confirmed the pluripotency of differentiation potentials into three germ layers (Fig. 1E). The differentiation potentials (pluripotency) of the iPSC clones, which were developed using the same method as the present study, was confirmed through teratoma formation assays (ectoderm: neural rosettes, endoderm: gut-like epithelia, and mesoderm: cartilage; Supp. Fig. S1).

**Table 1:** Clinical information.

Control or MSA	Cell line	Sex	Population	Age	Clinical subtype	Age of onset	*COQ2* mutation
Control	201B7	Female	Caucasian	36			None
	1201C1	Female	African	29			None
	HKC1	Male	Japanese	59			None
MSA_A	MSA_A26	Male	Japanese	61	MSA-C	44	Exon 7 c.[1159C > T]; [1178T > C] p.[R387*]; [V393A]
	MSA_A31						
	MSA_A34						
MSA_B	MSA_B1	Male	Japanese	56	MSA-C	54	None
	MSA_B2						
	MSA_B3						

**Figure 2:**
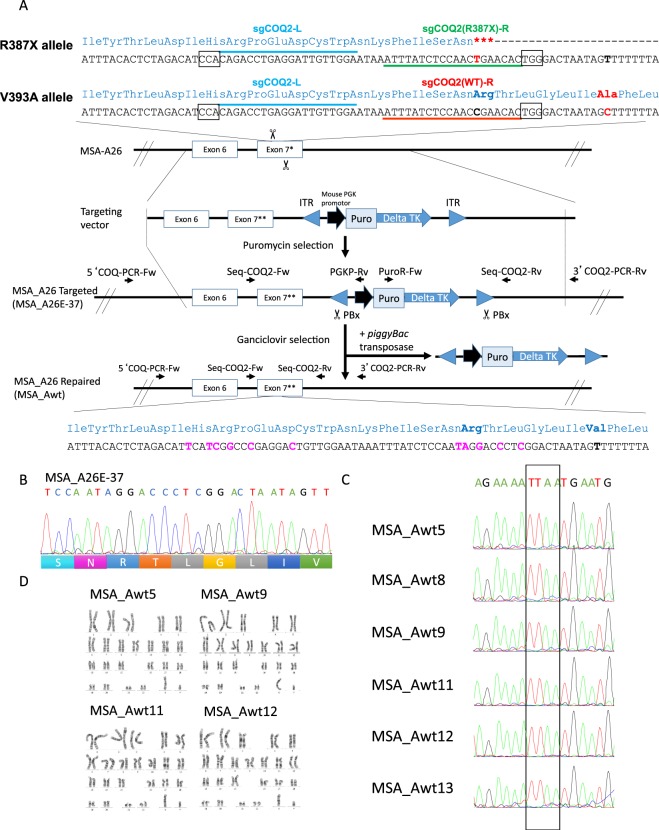
Site-specific gene correction. (**A**) Schematic overview for gene repair using CRISPR/Cas9 and *piggyBac* transposon methodology. Small arrows indicate primers for PCR and Sanger sequencing base screening to confirm the selected clones. PBx, excision-only *piggyBac* transposase; ITR, inverted terminal repeat; TK, thymidine kinase; Exon 7*, mutated exon 7; Exon 7**, donor exon 7. (**B**) Sanger DNA sequencing confirming of the gene edition. An iPSC clone (MSA_A26E-37) was identified as homozygous targeted. (**C**) Foot print analysis by sequencing. Sequences in the cassette-free repair clones indicated the exact repair after *piggyBac* excision. TTAA target sites are boxed. (**D**) Karyotype analysis. Four correctly repaired clones were confirmed normal karyotypes.

In addition, we generated isogenic iPSCs (MSA_Awt), wherein the *COQ2* mutation was corrected in MSA_A26 iPSCs using the CRISPR/Cas9 system (Fig. 2A). Puromycin-resistant human iPSC colonies obtained after electroporation were screened for proper targeting by nucleotide sequence analysis of the PCR products. One homozygously corrected clone (MSA_A26E-37) (Fig. 2B) was chosen for subsequent *piggyBac* excision experiments. *piggyBack* transposase transfection followed by ganciclovir selection yielded more than 18 drug-resistant colonies. Six of the 18 colonies were confirmed by nucleotide sequence analysis to show the presence of the characteristic “footprint” TTAA sequence at the site of transposase excision (Fig. 2C). Correctly repaired clones were chosen for karyotype analysis by the G-band staining method, and four clones were confirmed to show normal karyotypes (Fig. 2D). We used these three clones for further analysis (MSA_Awt5, Awt9, and Awt11).

### Induction of neural cells from MSA-iPSCs

**Figure 3:**
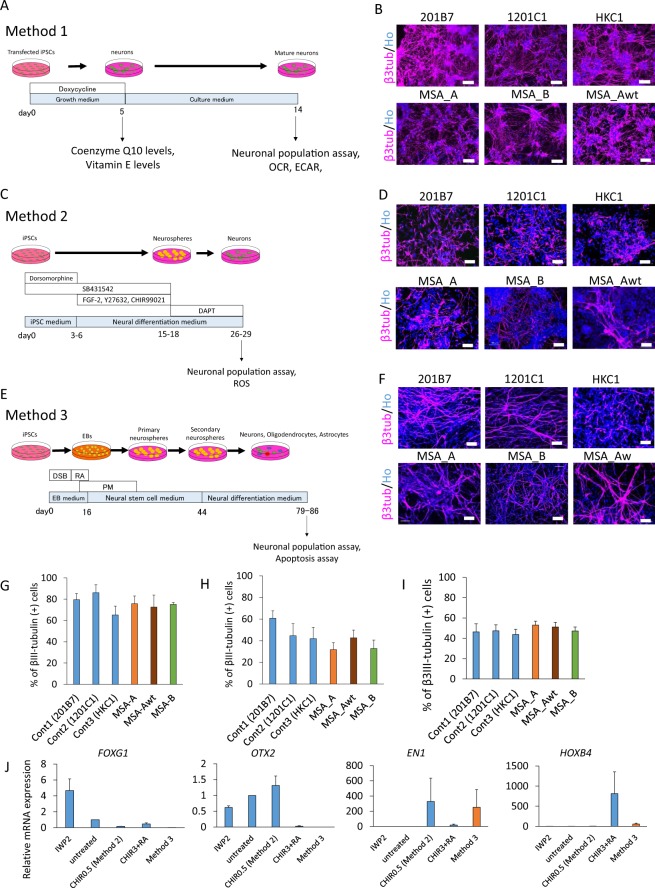
Neural cells differentiation from iPSCs. (**A**,**C**,**E**) Scheme of neuronal differentiation. (**A**) Method 1 (High efficiency induction of neurons), (**C**) Method 2 (Induction of mid-hindbrain neurons), (**E**) Method 3 (Induction of the three basic lineages of neural cells). DSB, dorsomorphine [D], SB431542 [S], and BIO [B]; RA, retinoic acid. (**B**,**D**,**F**) Analysis of the βIII-tubulin positive neurons. Representative images immunostaining (x20) and magnification for βIII-tubulin-positive cells. The scale bars represent 100 µm. (**B**) Method 1, (**D**) Method 2, (**F**) Method 3. (**G**,**H**,**I**) Differentiation efficiency in control, MSA_A, MSA_Awt, MSA_B-iPSCs. (**G**) Method 1 (on day 14 of iPSC differentiation), (**H**) Method 2 (on day 26–29 of iPSC differentiation), (**I**) Method 3 (on day 93 of iPSC differentiation). (**J**) qRT-PCR analysis of neurspheres from 1201C1 line for A-P marker expression (n = 3 independent experiments, mean ± SE).

All iPSCs were differentiated into neural lineage by three different methods (Fig. 3A,C,E); method 1 (high efficiency induction of neurons), method 2 (induction of mid-hindbrain neurons)^28^, and method 3 (induction of the three basic lineages of neural cells)^29^.

To examine the differentiation ratio, we performed immunocytochemistry for the neuronal marker, βIII-tubulin. Quantitative analysis of the expression of βIII-tubulin using IN Cell Analyzer (GE Healthcare) revealed that there were no significant differences in the differentiation ratio among controls, MSA_A, MSA_Awt and MSA_B iPSCs in all three neural induction models (Fig. 3B,D,F,G–I).

We next examined the expression of anteroposterior (A-P) markers in iPSC-derived neurospheres derived by method 2 (induction of mid-hindbrain neurons), using qRT-PCR (Fig. 3J). We induced the formation of neurospheres with the characteristics of four regions along the A-P axes by treatment with 2 µM IWP-2 (Sigma), 0.5 µM and 3 µM CHIR-99021 (CHIR) (Focus Biomolecules), and 1 µM retinoic acid (RA) (Sigma), as described in a previous report^28^. IWP-2-treated neurospheres expressed high levels of the forebrain marker *FOXG1*. The expression level of the forebrain/midbrain marker, *OTX2*, was high in untreated or 0.5 µM CHIR-treated neurospheres. *EN1*, which is expressed in the midbrain and the anterior hindbrain, was highly expressed in neurospheres treated with CHIR at a concentration of 0.5 µM. *HOXB4*, a marker of the posterior hindbrain and the spinal cord was expressed primarily in cultures exposed to 3 µM CHIR and 1 µM RA. These results confirmed the regional identity of neurospheres derived from iPSC lines and demonstrated that neurons generated by method 2 had characteristics of the midbrain and the hindbrain.

We also examined the expression of A-P markers in iPSC-derived neurospheres derived by method 3, using qRT-PCR (Fig. 3J). Expression levels of *FOXG1* and *OTX2* were low in neurospheres derived by method 3. *EN1* was highly expressed in these neurospheres, similar to the 0.5 µM CHIR-treated neurospheres (method 2). Expression level of *HOXB4* in neurospheres derived by method 3 was lower than that in 3 µM CHIR and RA-treated neurospheres. These results confirmed that neurons generated by method 3 also had characteristics of the midbrain and hindbrain, as well as of neurons generated from 0.5 µM CHIR-treated neurospheres (method 2) (Fig. 3J).

The neuronal cell subpopulations, differentiated using methods 1, 2, and 3, contained glutamatergic neurons (i.e., VGLUT1 and VGLUT2), GABAergic and glycinergic neurons (i.e., VGAT), and dopaminergic neurons (i.e., TH) (Supp. Fig. S2). There was no significant difference between the neurite lengths of MSA neurons and those of controls (Supp. Fig. S3A). To determine mitochondrial morphological changes in neural cells, we measured the area of the inner mitochondrial membrane (IMM). While changes in mitochontrial shape were not observed in neuronal cells from MSA patients, ranges of IMM area in MSA_A and MSA_B neurons were lower than those in control neurons (Supp. Fig. S3B,C).

Appropriate methods were chosen for each analysis as follows: Method 1, which provided high-efficiency neural induction (60–90%) (Fig. G), was used for analysing coenzyme Q10 levels, vitamin E levels, oxygen consumption rate and extracellular acidification rate. Method 2, which provided rapid neural induction of midbrain and hindbrain neurons^28^ that are preferentially affected in MSA, was used for analysing antioxidant activities. Method 3, which induced neurons and oligodendrocytes simultaneously^29^, was used for the apoptosis assay.

### Coenzyme Q10 levels and functional analysis of mitochondrial respiration and the antioxidative system in iPSC-derived neurons

**Figure 4:**
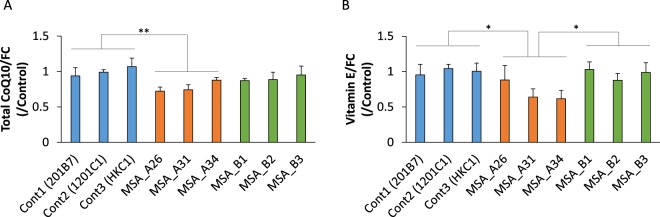
Intracellular levels of coenzyme Q10 and vitamin E in iPSC-derived neurons. (**A**) The levels of coenzyme Q10 was measured after adjustment for the free (unesterified) cholesterol level. The level of coenzyme Q10 of MSA_A neurons was significantly lower than those of control neurons. (**B**) The levels of vitamin E was measured after adjustment for the free (unesterified) cholesterol level. The level of vitamin E of MSA_A neurons was significantly lower than those of control and MSA_B neurons (n = 4 biological replicates per clone; mean ± SE, *p < 0.05, **p < 0.01, one-way ANOVA followed by Turkey’s post hoc test).

We measured intracellular coenzyme Q10 levels in iPSC-derived neurons (method 1) from patients with MSA and controls. Intracellular levels of coenzyme Q10 in iPSC-derived neurons from MSA_A were substantially lower than those in iPSC-derived neurons from controls (Fig. 4A). Intracellular coenzyme Q10 levels in MSA_B did not show significant differences compared to those in controls, a result consistent with the genotype containing the *COQ2* gene. Contrary to our expectations, however, intracellular level of coenzyme Q10 in MSA_Awt was still lower than those in controls (Supp. Fig. S4A). We also examined the expression of *COQ2* in iPSCs, using qRT-PCR, but MSA_A iPSCs did not show decreased expression levels of *COQ2* (Supp. Fig. S4C).

**Figure 5:**
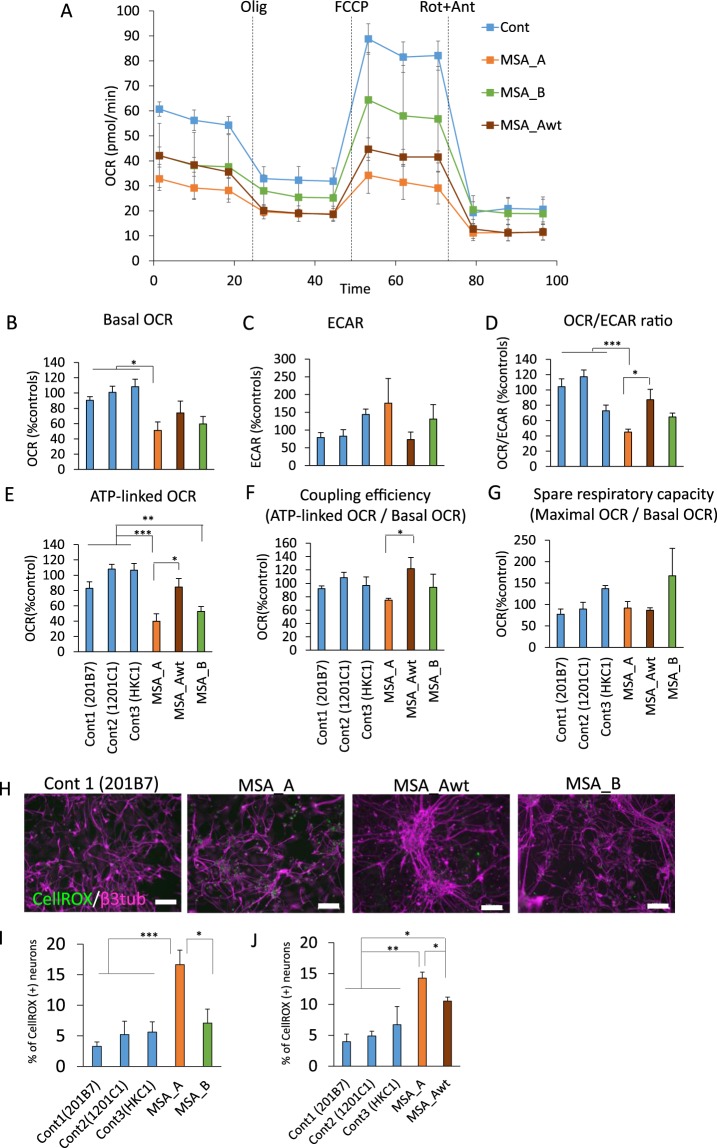
Functional analysis of mitochondrial respiration and antioxidative system in MSA iPSC-derived neurons. (**A**–**I**) Respiration ratios in MSA patient iPSC-derived neurons. (**A**) OCR profile in response to 1 µM oligomycin, 0.5–1 µM FCCP and 0.5 µM rotenone and antimycin A. For further details, see methods section. Decreased oxygen consumption was observed in MSA iPSC-derived neurons compared to control cells. Olig, oligomycin; Rot, rotenone; Ant, antimycin A; Cont, the average of Conts 1, 2 and 3. (**B**) Basal OCR; (**C**) ECAR; (**D**) OCR/ECAR ratio; (**E**) ATP-linked OCR; (**F**) Coupling efficiency; (**G**) Spare respiratory capacity (n ≥ 3 biological replicates per patient and subject; mean ± SE; *p < 0.05, **p < 0.01, ***p < 0.001, one-way ANOVA followed by Turkey’s post hoc test). (**H**) Representative images of immunocytochemistry for neurons using a marker for reactive oxygen species, CellROX. (**I**) CellROX positive cell population in MSA_A iPSC-derived neurons was significantly higher than that in control and MSA_B neurons (n ≥ 3 biological replicates per patient and subject; mean ± SE; *p < 0.05, ***p < 0.001, one-way ANOVA followed by Turkey’s post hoc test). (**J**) CellROX positive cell population in MSA_Awt iPSC-derived neurons was significantly lower than MSA_A neurons, but higher than control neurons (n ≥ 3 biological replicates per patient and subject; mean ± SE; *p < 0.05, **p < 0.01, one-way ANOVA followed by Turkey’s post hoc test).

Since coenzyme Q10 plays an essential role in the electron transport from complexes I and II to complex III in mitochondria, iPSC-derived neurons with *COQ2* mutations were predicted to have an impairment in the mitochondrial respiratory chain. Therefore, we determined the oxygen consumption rates (OCR, an indicator of mitochondrial respiration) and the extracellular acidification rate (ECAR, an indicator of glycolysis) in iPSC-derived neurons (method 1) from MSA_A, MSA_Awt, MSA_B, and control lines in response to the inhibitor of ATP synthase oligomycin, the proton ionophore carbonyl cyanide-p-trifluoromethoxyphenylhydrazone (FCCP) and the mitochondrial complex I inhibitors rotenone and antimycin A (Fig. 5A–G). We observed significantly reduced OCR, which reflects decreased basal OCR, as well as significantly reduced ATP-linked OCR (basal OCR minus the OCR measured after addition of oligomycin) and OCR/ECAR ratios in MSA_A iPSC-derived neurons compared with the values observed in controls (Fig. 5B,D,E). Notably, ATP-linked OCR was significantly lower not only in MSA_A but also in MSA_B iPSC-derived neurons. ATP-linked OCR, OCR/ECAR ratios and the ratio of the ATP-linked OCR against basal OCR (coupling efficiency) in MSA_Awt iPSC-derived neurons were significantly higher than those in MSA_A iPSC-derived neurons (Fig. 5E,F).

Since coenzyme Q10 also plays an essential role in antioxidant action, an increase in reactive oxygen species (ROS) was predicted in neurons from MSA patients with *COQ2* mutations compared to the levels observed in controls. Therefore, we examined ROS production using CellROX Green (Thermo Fisher Scientific) (Fig. J–L). The ratio of CellROX Green-positive neurons in the MSA_A iPSC-derived neurons was significantly higher than that in the controls and MSA_B iPSC-derived neurons. We observed no significant differences in the ratio between control and MSA_B iPSC-derived neurons (Fig. 5K). Moreover, the ratio of CellROX Green-positive neurons of MSA_Awt lines was significantly lower than that of MSA_A lines. On the other hand, we also observed significant differences between controls and MSA_Awt lines (Fig. 5L).

Next, we determined the levels of vitamin E, another antioxidant, whose level is correlated with coenzyme Q10 level (Fig. 4B). Since vitamin E represents the initial chain-breaking antioxidant during lipid peroxidation, reduced form of coenzyme Q10 (ubiquinol) appear to efficiently recycle the resultant vitamin E phenoxyl radical back to its biologically active reduced form^30^. Thus, a decrease in vitamin E levels in cells with coenzyme Q10 deficiency was predicted. In fact, vitamin E levels in iPSC-derived neurons from MSA_A were significantly lower than the levels in iPSC-derived neurons from controls and MSA_B. Contrary to our expectations, vitamin E levels in MSA_Awt iPSC-derived neurons were also significantly lower than the levels in iPSC-derived neurons from controls (Supp Fig. S4B).

### Apoptosis analysis under glucose-free conditions and coenzyme Q10 treatment

**Figure 6:**
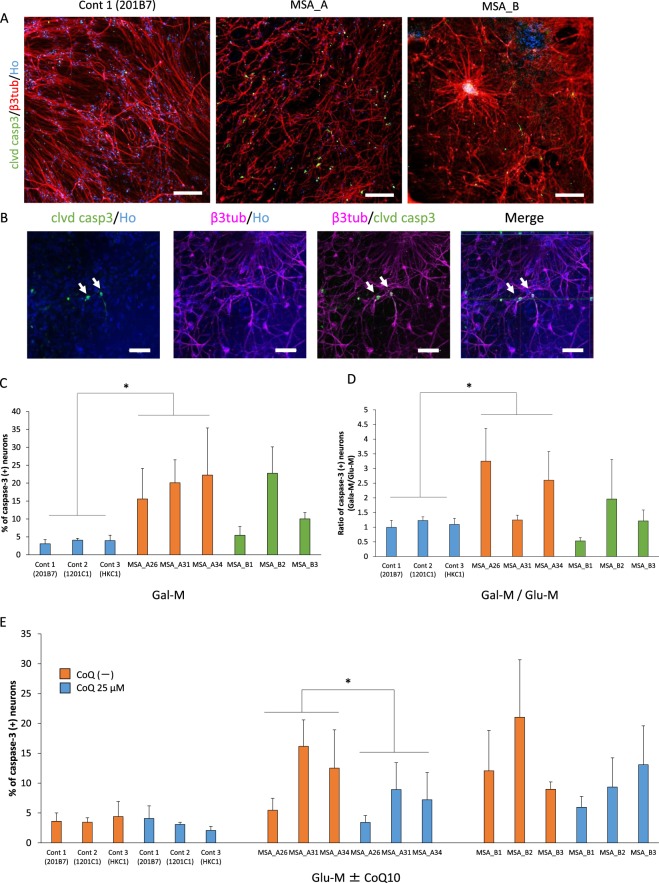
Apoptosis in MSA iPSC-derived neurons. (**A**,**B**) Representative images of immunocytochemistry for apoptotic neurons using a marker for apoptosis, cleaved-Caspase 3. Neurons that were βIII-tubulin/cleaved Caspase 3-double-positive were considered apoptotic neurons. Clvd casp3, cleaved-Caspase 3. (**A**) The scale bars represent 200 µm. (**B**) Images of MSA_A line. The scale bars represent 50 µm. (**C**) Quantitative data of the ratio of cleaved-Caspase 3-positive cells in βIII-tubulin-positive neurons in glucose-free medium (Gal-M) (n = 3 biological replicates per clone; mean ± SE, *p < 0.05, Dunnett’s test). (**D**) Effect of glucose-free condition on the ratio of cleaved-Caspase 3-positive cells in βIII-tubulin-positive neurons. Glu-M, normal glucose-containing medium (n = 3 biological replicates per clone; mean ± SE, *p < 0.05, Dunnett’s test). (**E**) Effect of coenzyme Q10 (CoQ10) treatment on the ratio of cleaved-Caspase 3-positive cells in βIII-tubulin-positive neurons in normal condition (n = 3 biological replicates per clone; mean ± SE, *p < 0.05, paired *t*-test).

Neurodegeneration is observed post-mortem in the striatum and midbrain of MSA patients. This finding suggested that neurons of MSA patients demonstrated cellular vulnerability^4^. Therefore, we pursued the cellular vulnerability of MSA-derived neurons using immunocytochemistry for cleaved-Caspase 3 (Fig. 6A,B). To examine the relationship between neuronal apoptosis and an impairment of the mitochondrial respiratory chain, we used a glucose-free medium, which forced the cells to rely heavily on oxidative phosphorylation instead of glycolysis to generate ATP, as a stress condition. The replacement of glucose with galactose in the culture medium to force cells to rely more heavily on oxidative phosphorylation for ATP formation has been used to determine underlying metabolic defects in several cell lines^31^. In the present study, the mean ratios of cleaved-Caspase 3 positive neurons in MSA lines under the stress condition (glucose-free medium) tended to be higher than those in control lines (MSA_A *p* = 0.01, MSA_B *p* = 0.07) (Fig. 6C). Moreover, the relative change in the apoptotic cell population under stress conditions versus control conditions (normal glucose-containing medium) was much larger in MSA_A neurons than in control neurons (Fig. 6D). On the other hand, the expression level of cleaved-Caspase 3 in rescued group (MSA_Awt) was still higher than those in controls (Supp. Fig. S6).

In addition, in order to determine if the increase in apoptosis observed in MSA lines was the result of decreased coenzyme Q10 levels, ratios of cleaved-Caspase 3 positive neurons with coenzyme Q10 supplementation in the control condition were measured. The ratio of apoptotic neurons in MSA_A lines with coenzyme Q10 supplementation was significantly lower than that in cell lines without supplementation (Fig. 6E).

To confirm that the cleaved-Caspase 3 antibody indeed reflected neuronal apoptosis, we analysed the immunocytochemistry of cleaved-Caspase 3 after treatment with hydrogen peroxide (H_2_O_2_), which induces apoptosis^32–34^. Incubation with 0–1 mM H_2_O_2_ caused a concentration-dependent increase in cleaved-Caspase 3-positive neurons and in LDH activities (Supp. Fig. S5). The results suggested that the cleaved-Caspase 3 antibody indeed reflected neuronal apoptosis.

## Discussion

In the present study, we established human iPSCs from two patients with MSA, one of whom carried compound heterozygous mutations in the *COQ2* gene. In addition, we differentiated iPSCs into patient-derived neurons to observe the disease phenotype of MSA *in vitro*. The model demonstrated increased neuronal apoptosis.

MSA-derived neurons with *COQ2* mutations showed decreased levels of coenzyme Q10 and cellular dysfunctions attributable to decreased coenzyme Q10 levels such as impairment of the mitochondrial respiratory chain and antioxidant actions. We also examined the expression of *COQ2* in iPSCs, using qRT-PCR, but MSA_A iPSCs did not show decreased expression level of *COQ2*. It is suggested that the expression of *COQ2* was not decreased while there was COQ2 dysfunction in MSA_A cells. We observed significantly decreased basal OCR, ATP-linked OCR, and OCR/ECAR ratios in MSA_A iPSC-derived neurons, which suggests that iPSC-derived neurons with *COQ2* mutations have an impairment in the mitochondrial respiratory chain. In addition, the ratios of CellROX Green-positive neurons in iPSC-derived neurons from MSA_A were significantly higher than those in iPSC-derived neurons from controls and MSA_B. These results suggest that compound heterozygous mutations of *COQ2* increased cellular oxidative stress. Interestingly, vitamin E levels in iPSC-derived neurons from MSA_A were significantly lower than those in iPSC-derived neurons from controls and MSA_B. It is suggested that decreased levels of coenzyme Q10 and vitamin E increased cellular oxidative stress production in iPSC-derived neurons from MSA_A.

The result obtained by coenzyme Q10 supplementation in the apoptosis assay supports the causal relationship between neuronal apoptosis and coenzyme Q10 deficiency in neurons from iPSCs of MSA patients carrying *COQ2* mutations. Furthermore, the result obtained from the glucose-free assay suggested that neuronal apoptosis in MSA with *COQ2* mutations was linked to the impairment of the mitochondrial respiratory chain. It was suggested that when glycolysis was suppressed using a glucose-free condition, the necessary ATP production was not achieved in MSA_A with an impaired mitochondrial respiratory chain, and the vulnerability of neurons was increased. This hypothesis was supported by the result of the OCR assay, which suggested that OCR in MSA_A neurons was lower than that in control neurons in a glucose-containing condition.

From a therapeutic viewpoint, supplementation with coenzyme Q10 may be helpful in treating MSA, particularly for patients with *COQ2* variants. In our previous report, although ubiquinol improved mitochondrial oxidative metabolism in the brain of MSA_A, the scores of clinical rating scales and the brain MRI results remained unchanged, presumably because the patient was at an advanced stage of MSA^25^. However, the present study showed decrease in apoptosis in iPSC-derived neurons upon coenzyme Q10 supplementation, and the result suggested the therapeutic potential of coenzyme Q10 for patients in the early phase of MSA with *COQ2* mutation.

Next, we introduced site-specific corrections into an iPSC clone derived from the MSA patient carrying the *COQ2* mutation using CRISPR/Cas9. The ratio of ROS marker positive neurons decreased, and ATP-linked OCR, OCR/ECAR ratio, and coupling efficiency increased in the isogenic lines. These results suggested the causal relationship between *COQ2* mutations and cellular dysfunctions attributable to decreased coenzyme Q10 levels. Interestingly, the ratio of ROS marker positive neurons in the isogenic lines was still higher than that in control lines. This suggested that the cellular dysfunction was not alleviated completely. The ratio of apoptosis marker positive neurons derived from isogenic lines was also still higher than that from control lines. Thus, unidentified additional factors may have led to increased ROS and apoptosis in MSA_A. Contrary to our expectations, the coenzyme Q10 level in neurons from the isogenic lines (MSA_Awt) was still lower than those from control lines. The mechanisms underlying the decreased level of coenzyme Q10 in MSA_Awt neurons remain to be elucidated. Some studies have reported that patients with MSA, not only patients carrying the *COQ2* mutation but also those not carrying the mutation, had significantly lower levels of coenzyme Q10 in blood and frozen autopsied cerebellar tissues than the controls^20,22,23^. These findings strongly support the idea that coenzyme Q10 insufficiency plays a role in the pathogenesis of MSA even in patients not carrying *COQ2* mutations.

Some of the assays in MSA_B neurons also yielded partially unexpected results; MSA_B neurons showed a slight impairment of the mitochondrial respiratory chain, while they did not show significant decreases in coenzyme Q10 levels and increases in oxidative injury. Decreased coenzyme Q10 levels and increased oxidative injury were previously reported in post-mortem brains and other tissues of MSA patients, regardless of the *COQ2* genotype^20–23,35^. In addition, the increase in the apoptotic cell population induced by the glucose-free condition in MSA_B neurons was not significantly different from that observed in control neurons. This result suggested that ATP production was achieved in MSA_B neurons in the glucose-free condition as well as in control neurons.

To clarify the role of coenzyme Q10 in the pathogenesis of MSA, further analysis is needed. Previous studies have shown that there were large differences among individuals in levels of coenzyme Q10, and there were MSA patients whose coenzyme Q10 levels were equal to or higher than those of controls^20,22,23^. Further analysis by increasing the number of MSA patients and the assessment of cellular phenotypes in isogenic iPSCs with *COQ2* mutation using genome editing systems on control iPSCs and MSA without *COQ2* mutation will be needed.

The current study demonstrated the genotype-phenotype causal relationship between *COQ2* mutations and dysfunctions of the mitochondrial respiratory chain and antioxidant action in neurons differentiated from iPSCs derived from MSA patients, suggesting the therapeutic potential of coenzyme Q10 for MSA patients with *COQ2* mutation. Interestingly, MSA patient-derived neurons without *COQ2* mutation also showed some decrease in mitochondrial respiratory functions. The underlying mechanism of this observation should be clarified in the future to improve our understanding of this disease.

In addition, we analysed the accumulation of alpha-synuclein in MSA iPSC-derived oligodendrocytes and neurons by immunocytochemistry. However, we could not detect differences between MSA patients cells and healthy control cells. Because MSA is a disease with middle-age onset, the cellular senescence achieved in the present cultures might be insufficient because of the short timeframes. Our results suggested that caspase 3 was activated before α-synuclein accumulated in neurons derived from MSA patients. It is not clear how α-synuclein relates to the pathogenesis of MSA, but the present results might be useful in investigating the neuronal apoptosis mechanism in MSA.

## Methods

### Isolation of human PBMCs and generation of induced pluripotent stem cells (iPSCs)

Human PBMCs from the blood of two Japanese male patients were used to establish MSA_A iPSCs and MSA_B iPSCs. Human PBMCs from a 59-year-old healthy male were used to establish HKC1-iPSCs (control 3). Additional control cell lines used in this study included 201B7 (control 1, established from human dermal fibroblasts (HDFs) from the dermis of a 36-year-old Caucasian female) and 1201C1 (control 2, established from human PBMCs of a 29-year-old African American female) (Table 1). MSA_A, MSA_B and HKC1-iPSC clones were established using episomal plasmid vectors carrying reprogramming genes (*OCT4*, *SOX2*, *KLF4*, *L-MYC*, *LIN28*, *dominant-negative p53*) into human PBMCs as described previously^27^ and evaluated based on the expression of pluripotent stem cell markers and the elimination of transgenes. Three clones for each group were used for further analysis: control (201B7, 1201C1 and HKC1), MSA_A (A26, A31, A34), and MSA_B (B1, B2, B3). Data from the cell lines of patients are expressed as the average of the three clones. All experimental procedures for iPSC production were approved by Keio University School of Medicine Ethics committee (approval number, 20080016) and University of Tokyo Ethics committee (approval number, G2876-(2)). All methods were performed in accordance with the relevant guidelines and regulations of the institutions. Informed consent was obtained from all subjects.

### *In vitro* differentiation into neural cells

For establishing single-step neuronal-inducible iPSC lines for method 1 (high efficiency induction of neurons), we used the following vectors: PB-CAG-rtTA3G-IH, PB-TET-PH-lox66FRT-NEUROG2^36^ and pCMV-HyPBase-PGK-Puro^37^, which were kindly provided by Dr. Minoru S. H. Ko and Dr. Kosuke Yusa, respectively. iPSCs were cultured in StemFit AK02N on 6-well plates with iMatrix-511 coating, and 1–2 × 10^5^ of input iPSCs were transfected with 1.5 µg of expression vectors for HyPBase, rtTA3G and NGN2 were transfected by 4.5 µl Gene Juice (Novagen). Colonies were exposed with StemFit AK02N containing 150 µg/ml hygromycin (Wako) and 0.2–1.0 µg/ml puromycin (Sigma), and counted up 5–9 days after planting. To induce the development of neurons, transfected hiPSCs were seeded onto tissue culture dishes coated with poly-L-ornithine (Thermo Fisher Scientific) and iMatrix-511 (D = 0). The cells were cultured in serum-free growth medium (medium hormone mix; MHM)^38^ containing B27 supplement (Thermo Fisher Scientific), Y27632 and 3 µM DAPT (Sigma). Approximately 20 µg/ml doxycycline (Wako) was added on D = 0 to induce transgene expression. On D = 5, the medium was replaced with culture medium (MHM containing 1x B27, 20 ng/ml brain derived neurotrophic factor (BDNF) (R & D), 10 ng/ml glial cell line-derived neurotrophic factor (GDNF) (Alomone labs), 200 µM L-ascorbic acid (Sigma), 100 µM dibutyryl cyclic adenosine monophosphate (AMP) (Sigma) and 3 µM DAPT) (Fig. 3A). The medium was changed every 3 days.

Neuronal induction of method 2 (induction of mid-hindbrain neurons) was performed as described previously^28^ (Fig. 3C). Briefly, iPSCs were pre-treated for three days with 3 μM SB431542 (Sigma), and 3 μM dorsomorphin (Santa Cruz). They were then dissociated and seeded at a density of 20 cells/μL in MHM with selected growth factors and inhibitors under conditions of 4% O_2_/5% CO_2_. The growth factors and inhibitors included 20 ng/ml fibroblast growth factor 2 (FGF-2) (PeproTech), 1 × B27, 2 μM SB431542, and 10 μM Y-27632. Defining the day on which the neurosphere culture was started as D = 0, CHIR-99021 (CHIR) was included in the neurosphere culture. On day 12, neurospheres were replated on dishes coated with poly-ornithine and growth-factor-reduced Matrigel and cultured under conditions with 5% CO_2_. The medium was changed to MHM supplemented with 1 × B27 and 1 μM DAPT.

To determine the expression of A-P markers in the iPSC-derived neurospheres, the following additives were included in the neurosphere culture: 2 µM IWP-2, 0.5–3 µM CHIR-99021 (CHIR), and 1 µM retinoic acid (RA).

Neural differentiation of method 3 (induction of the three basic lineages of neural cells) was also performed as previously described^29^ (Fig. 3E). Briefly, iPSC colonies were detached from feeder layers and cultured in suspension as EBs for approximately 16 days in non-treated culture dishes. EBs were then dissociated into single cells with TrypLE Select and the dissociated cells were cultured in suspension in MHM for 14 days to allow the formation of neurospheres. Neurospheres were passaged by dissociation into single cells followed by culture in the same manner. The secondary neurospheres were used for analysis. For terminal differentiation, undissociated neurospheres were allowed to adhere to poly-L-ornithine and growth-factor-reduced Matrigel-coated coverslips and cultured for five weeks until analyses.

### Neuronal population assay

The iPSC-derived neurons of each method were fixed and subjected to immunocytochemical analyses using the anti-βIII-tubulin antibody. βIII-tubulin-positive cells were counted using IN Cell Analyzer 6000. Neuronal population was defined as the number of βIII-tubulin-positive cells divided by the number of nuclei.

### Coenzyme Q10 and vitamin E levels in iPSC-derived neurons

Levels of coenzyme Q10 (ubiquinone-10 and ubiquinol-10), vitamin E and free (unesterified) cholesterol in iPSC-derived neurons (method 1) were measured using high-performance liquid chromatography^39^.

### Determination of oxygen consumption rates (OCR) and extracellular acidification rate (ECAR)

Mitochondrial OCR and ECAR in cell cultures were measured using an XF24-3 Extracellular Flux Analyzer (Seahorse Bioscience).

The transfected iPSCs (method 1) were seeded at a density of 2 × 10^4^ cells/well in XF^e^24-well cell culture microplate (Seahorse Bioscience) and cultured in the growth medium with doxycycline at 37 °C in 5% CO_2_ atmosphere. Five days after seeding cells, the medium was replaced with culture medium without doxycycline, and cells were cultured for nine days.

The medium was replaced with Seahorse XF media (Seahorse Bioscience), supplemented with 25 mM glucose and 1 mM sodium pyruvate (Sigma), and the plates were pre-incubated in a CO_2_-free incubator at 37 °C for one hour for equilibration and thereafter processed in the XF analyser for OCR and ECAR analysis. OCR and ECAR were recorded three times and followed by sequential injections of oligomycin (1 µM), FCCP (0.5 or 1 µM), and antimycin A and rotenone (0.5 µM) into each well. Basal OCR was calculated by subtracting the OCR values obtained after the addition of rotenone and antimycin A from the OCR values of the third measurement of the experiment. The OCR/ECAR ratio was calculated by averaging the ratio of OCR against ECAR obtained during first three readings of the experiment. ATP-linked OCR was calculated by subtracting the OCR values obtained after the addition of oligomycin from basal OCR. Coupling efficiency was calculated by the ratio of ATP-linked OCR against basal respiration. Spare respiratory capacity was calculated using the ratio of maximal OCR, which was calculated by subtracting the OCR values obtained after the addition of rotenone and antimycin A from the OCR values obtained after the addition of FCCP, against basal OCR. Since it is difficult to perform the assay in many clones, we chose one clone each for MSA_A, MSA_Awt, and MSA_B (MSA_A26, MSA_Awt5, and MSA_B3) to perform the assay effectively. 4 out of 6 clones (201B7, 1201C1, HKC1, MSA_A26, MSA_Awt5, and MSA_B3) were tested simultaneously, and we changed the combination of the 4 clones for each experiment. Data was relativized to the ratios average of the controls for each condition (Fig. 5B–G).

### Analysis of antioxidant activities

We estimated oxidant levels in control and MSA iPSC-derived neurons (method 2) using CellROX Green, a fluorogenic probe for measuring oxidative stress in live cells. Adherent cells were incubated with 5 µM CellROX for 30 min at 37 °C in the dark and washed once with PBS. iPSC-derived neurons were fixed with 4% paraformaldehyde and subjected to immunocytochemical analyses using the anti-βIII-tubulin antibody. βIII-tubulin-positive cells or βIII-tubulin/CellROX-double-positive cells were counted using IN Cell Analyzer 6000. Percentages of CellROX-positive neurons were defined as the number of βIII-tubulin- and CellROX-double-positive cells divided by the number of βIII-tubulin-positive cells.

### Apoptosis analysis with glucose-free conditions and coenzyme Q10 treatment

To demonstrate the vulnerability of neurons derived from MSA patients, expression of cleaved-Caspase 3, which is a marker for apoptosis, was measured. iPSC-derived neurons were fixed and subjected to immunocytochemical analyses using the anti-βIII-tubulin antibody and anti-cleaved Caspase 3 antibody. βIII-tubulin-positive cells or βIII-tubulin/cleaved-Caspase 3-double-positive cells were counted using IN Cell Analyzer 6000. Cell vulnerability was defined as the number of βIII-tubulin- and cleaved-Caspase 3-double-positive cells divided by the number of βIII-tubulin-positive cells.

For the glucose-free condition assay, neurospheres (method 3) were grown in an adherent culture for six weeks before they were incubated for seven days in normal glucose-containing medium (contained 0.6% glucose) or glucose-free medium (contained 0.6% galactose).

For experiments analysing the therapeutic efficacy of coenzyme Q10, neurospheres were grown in an adherent culture for five weeks before they were incubated in fresh medium supplemented with 25 µM coenzyme Q10 (Nisshin Pharma) for one week. After the supplementation, neurospheres were incubated for seven days in normal glucose-containing medium.

For experiments analysing the efficacy of H_2_O_2_, 1201C1 secondary neurospheres of method 3 grown in 48-well plates were incubated with differentiation medium for two weeks. To force apoptosis, the cells were incubated with 0–1 mM H_2_O_2_ (Wako) for 24 h.

## Electronic supplementary material

Supplementary Information

## Data Availability

The data that support the findings of this study are available from the corresponding author upon reasonable request.
