# Supplementary material for: The pathogenesis linked to coenzyme Q10 insufficiency in iPSC-derived neurons from patients with multiple-system atrophy
Source: Sci Rep. 2018 Sep 21;8:14215. doi: 10.1038/s41598-018-32573-1 (PMC6155102; doi:10.1038/s41598-018-32573-1)
Supplement: Supplementary file 1 — Supplementary Information [file 41598_2018_32573_MOESM1_ESM.pdf]

The pathogenesis linked to coenzyme Q10 insufficiency in iPSC-derived neurons from patients with multiple-system atrophy

Fumiko Kusunoki Nakamoto [1,2], Satoshi Okamoto [2], Jun Mitsui [1], Takefumi Sone [2], Mitsuru Ishikawa [2], Yorihiro Yamamoto [3], Yumi Kanegae [4], Yuhki Nakatake [5], Kent Imaizumi [2], Hiroyuki Ishiura [1], Shoji Tsuji [1] and Hideyuki Okano [2,*]

[1]Department of Neurology, University of Tokyo, School of Medicine, Hongo, Bunkyo-ku, Tokyo, 113-8655, Japan.

[2]Department of Physiology, Keio University School of Medicine, Shinanomachi, Shinjuku-ku, Tokyo, 160-8582, Japan.

[3]School of Bioscience and Biotechnology, Tokyo University of Technology, Katakuramachi, Hachioji City, Tokyo, 192-0914, Japan.

[4]Research Center for Medical Science, Jikei University School of Medicine, Nishi-shinbashi, Minato-ku, Tokyo, 105-8461, Japan.

[5]Department of Systems Medicine, Keio University School of Medicine, Shinanomachi, Shinjuku-ku, Tokyo, 160-8582, Japan.

* Correspondence: hidokano@a2.keio.jp (H.O.)

Supp Figure S1

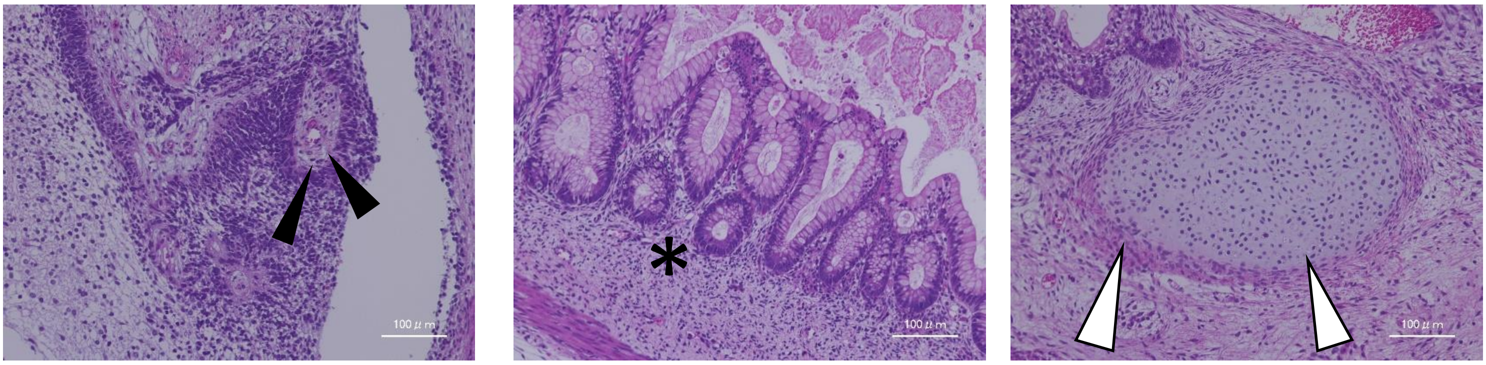

ectoderm endoderm mesoderm

Supp Figure S2

Method 1

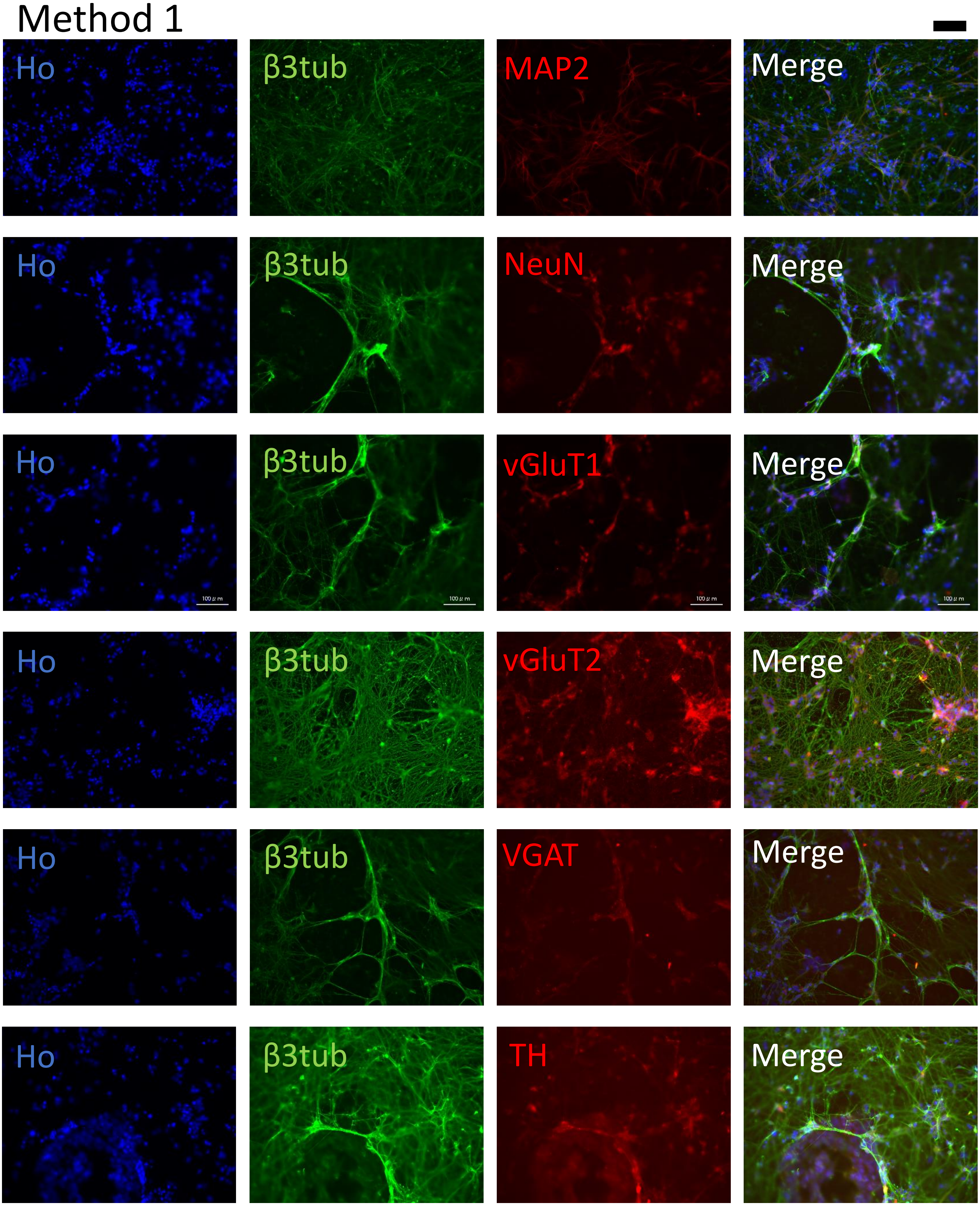

Method 2

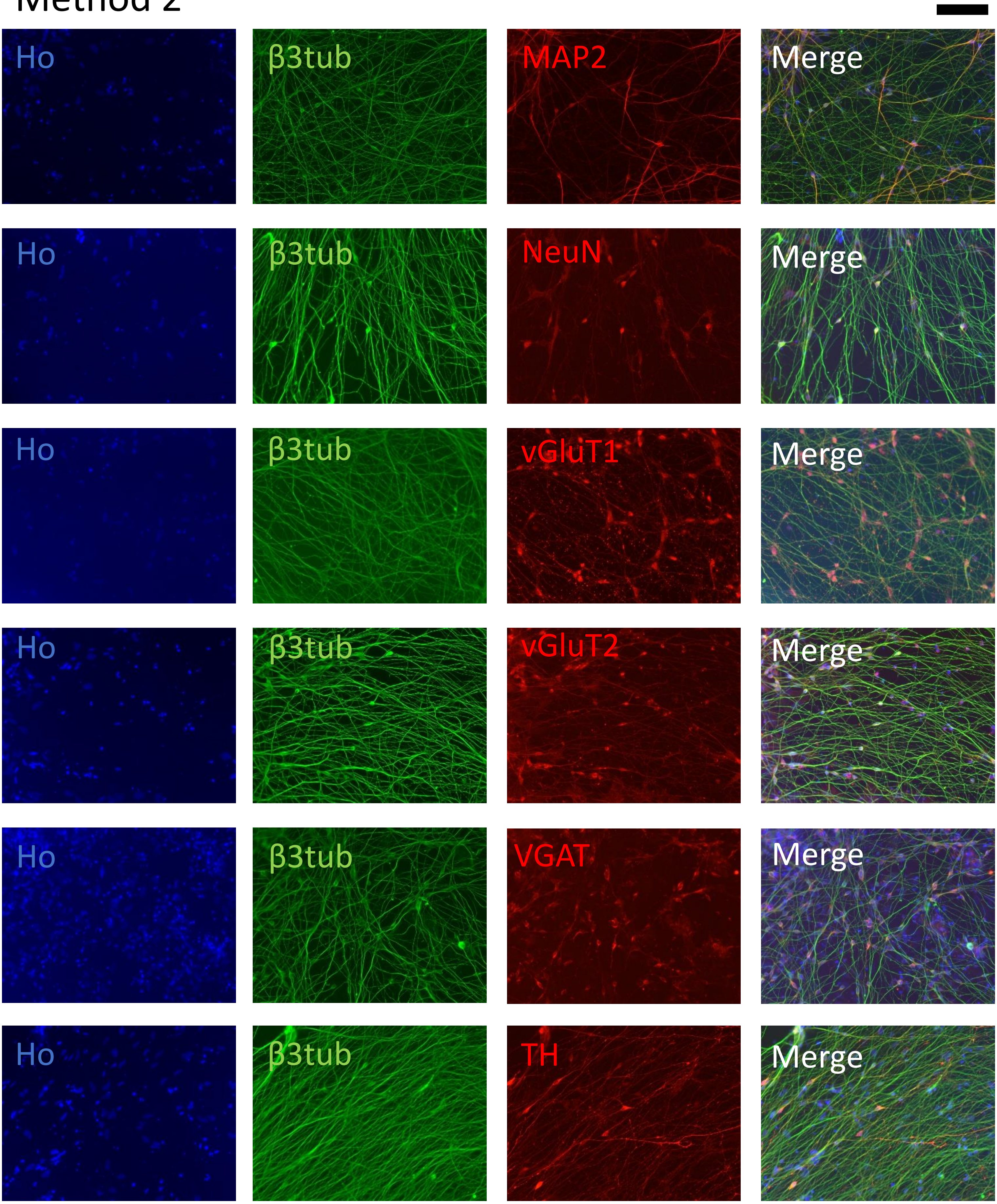
Ho
β3tub
MAP2
Merge
Ho
β3tub
NeuN
Merge
Ho
β3tub
vGluT1
Merge
Ho
β3tub
vGluT2
Merge
Ho
β3tub
VGAT
Merge
Ho
β3tub
TH
Merge

Method 3

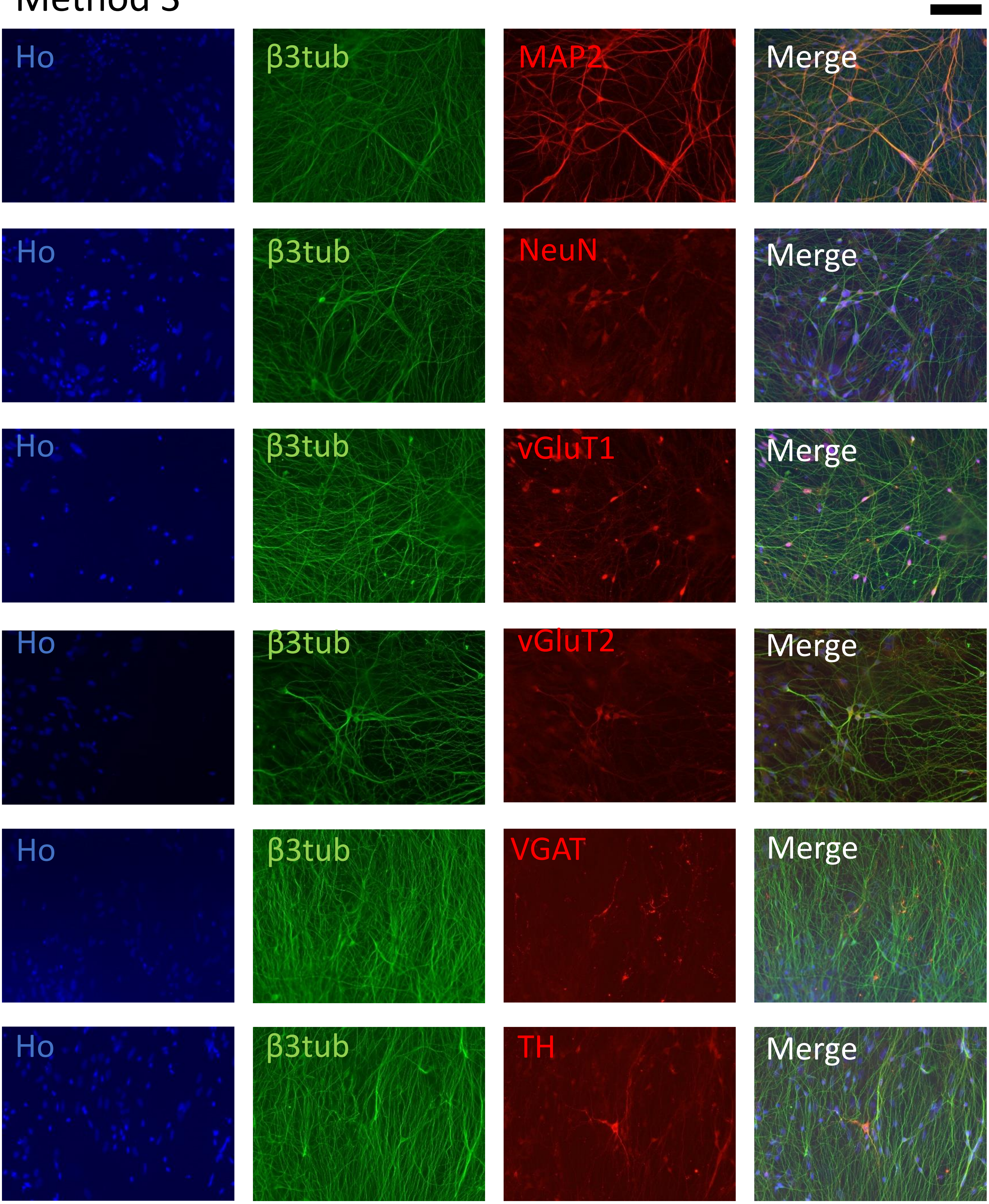
Ho
β3tub
MAP2
Merge
Ho
β3tub
NeuN
Merge
Ho
β3tub
vGluT1
Merge
Ho
β3tub
vGluT2
Merge
Ho
β3tub
VGAT
Merge
Ho
β3tub
TH
Merge

Supp Figure S3

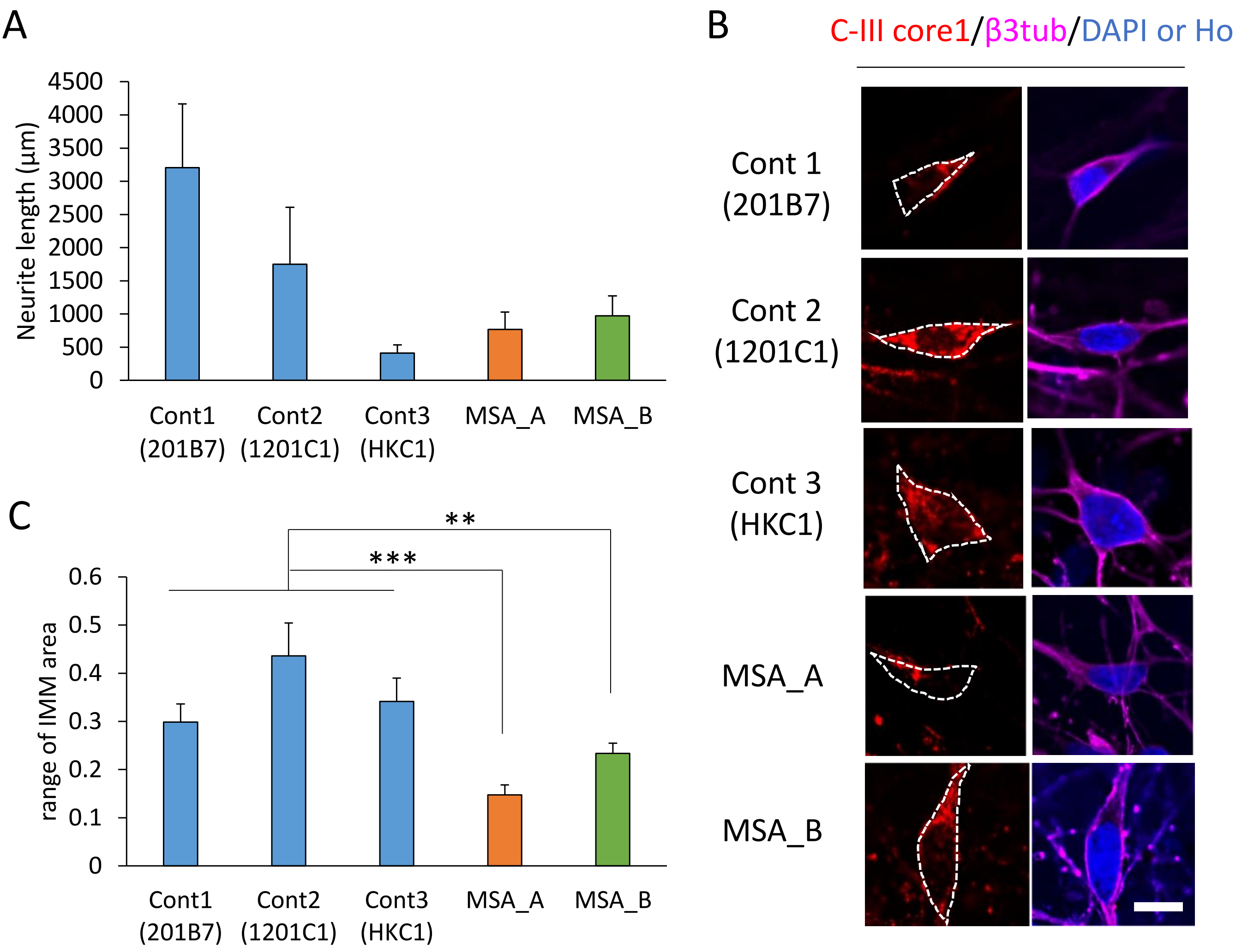

Supp Figure S4

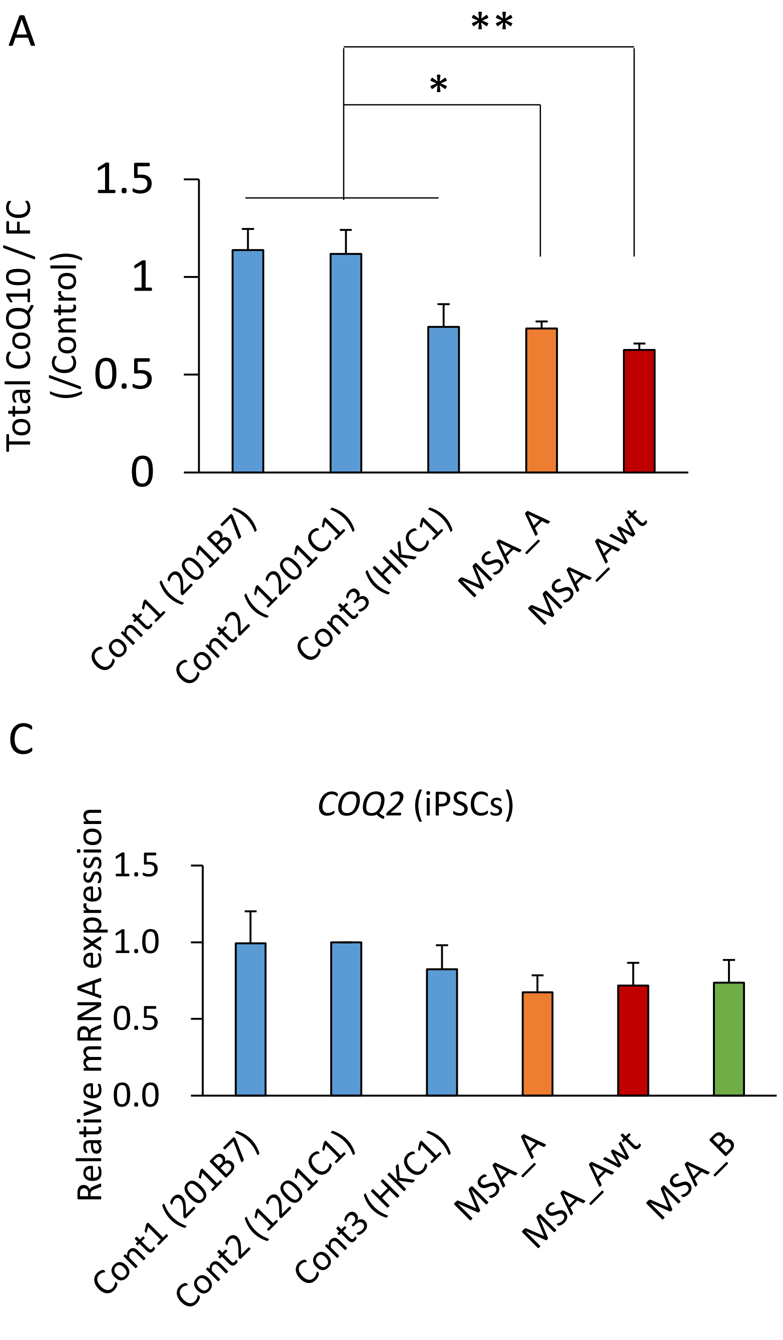

Supp Figure S5

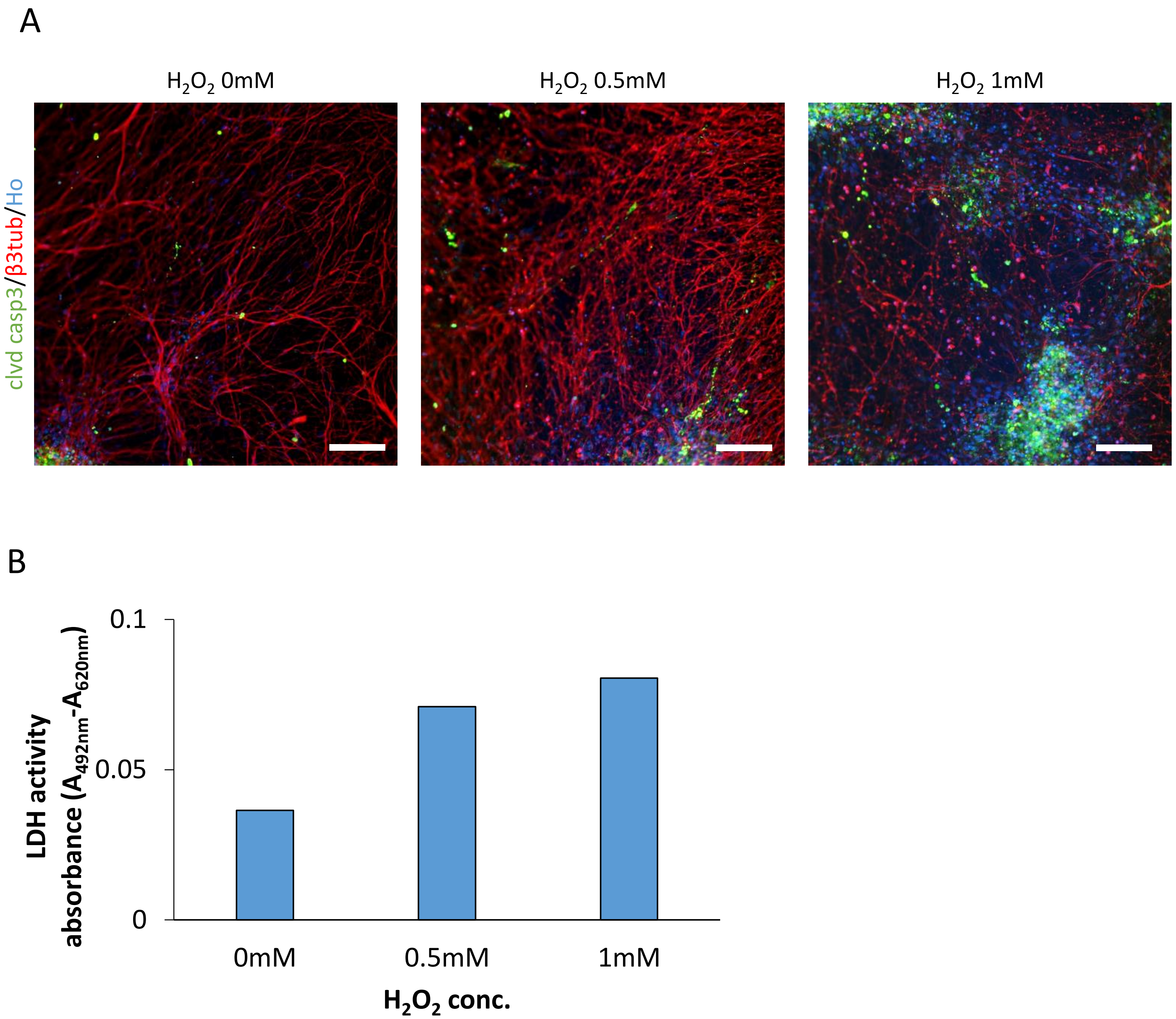

Supp Figure S6

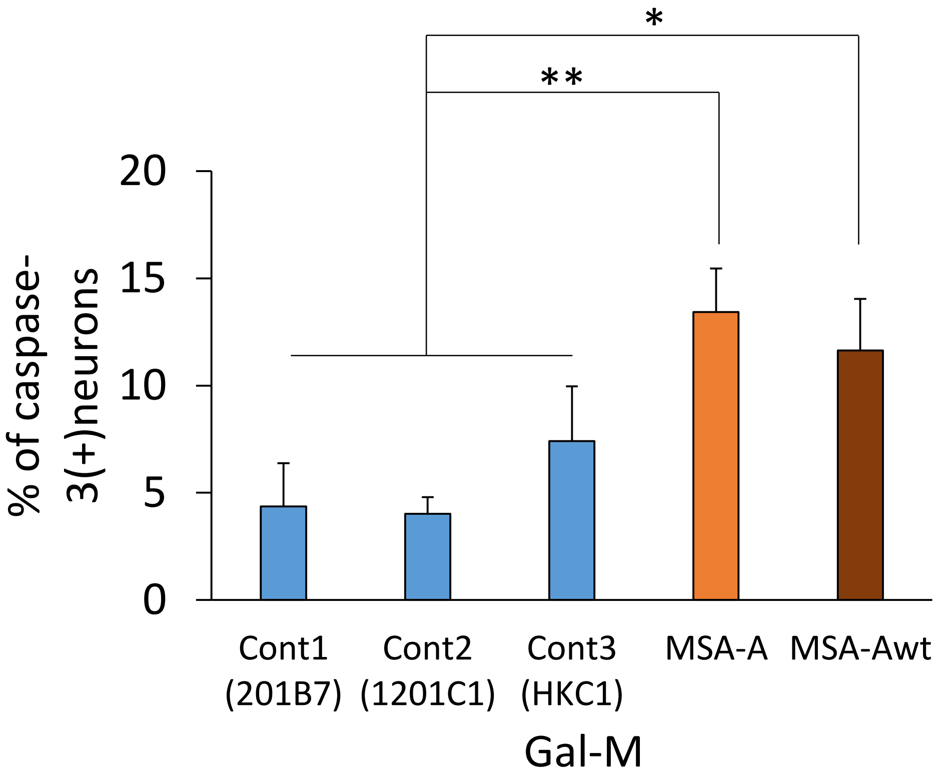

Figure Legends

Supp. Figure S1. Representative H&E staining of teratomas derived from established another MSA iPSC clone.

Teratomas were formed via the injection of undifferentiated iPSCs into the testes of NOD/SCID mice. Arrowhead, neural rosette. Asterisks, gut-like epithelium. Open arrowhead, cartilage.

Supp. Figure S2. Representative image of immunocytochemistry for βIII-tubulin and other neural markers (MAP2, NeuN, VGLUT1, VGLUT2, VGAT, TH).

(A) Method 1; (B) Method 2; (C) Method 3**.** The scale bars represent 100 µm. Ho, Hoechst33258; β3tub, βIII-tubulin.

Supp. Figure S3. Neurite outgrowth and inner mitochondrial membrane marker in MSA iPSC-derived neurons.

(A) Quantitative data of the neurite length of βIII-tubulin-positive neurons

(B) Double labeling for the inner mitochondrial membrane (IMM) marker, ComplexIII coreI (C-III core1; red) and βIII-tubulin (magenta) of cont 1 (201B7), cont 2 (1201C1), cont 3 (HKC1) and MSA (MSA_A and MSA_B) iPSC-derived neurons (method 3). The scale bar represents 10 µm.

(C) The ranges of IMM area in MSA_A and MSA_B neurons were lower than those in control neurons (n≥3 biological replicates per patient and subject; mean ± SE, ***p* < 0.01, *** *p* <0.001, Dunett's test).

Supp.Figure S4. Intracellular levels of coenzyme Q10 and vitamin E and expression level of *COQ2*.

(A) The level of coenzyme Q10 in MSA_Awt was still lower than those in controls.

(B) The level of vitamin E of MSA_Awt neurons was significantly lower than those of control neurons (N=4 biological replicates per clone; mean ± SE, **p* < 0.05, ** *p* <0.01, **** *p* <0.0001, one-way ANOVA followed by Turkey's post hoc test).

(C) qRT-PCR analysis of iPSCs for *COQ2* (n=4 independent experiments, mean ± SE).

Supp. Figure S5. Confirmation of the cleaved-Caspase 3 antibody as a neuronal apoptosis marker.

(A) Representative images of immunocytochemistry for apoptotic neurons after H_2O_2 supplementation. Clvd casp3, cleaved-Caspase 3. The scale bars represent 50 µm.

(B) Neurospheres of method 3 were cultured for 24h in fresh media with 0–1 mM H_2O_2. LDH activities were measured in the media.

Supp. Figure S6. Apoptosis in MSA iPSC-derived neurons.

The expression level of cleaved-Caspase 3 in rescued group (MSA_Awt) was still substantially higher than those in controls (n≥3 independent experiments; mean ± SE; *p < 0.05, **p < 0.01, Dunnett's test).

Methods

Isolation of human PBMCs and generation of induced pluripotent stem cells (iPSCs).

Human PBMCs from the blood of two Japanese male patients were used to establish MSA_A iPSCs and MSA_B iPSCs. Human PBMCs from a 59-year-old healthy male were used to establish HKC1-iPSCs (control 3). Additional control cell lines used in this study included 201B7 (control 1, established from human dermal fibroblasts (HDFs) from the dermis of a 36-year-old Caucasian female) and 1201C1 (control 2, established from human PBMCs of a 29-year-old African American female) (Table 1). MSA_A, MSA_B and HKC1-iPSC clones were established using episomal plasmid vectors carrying reprogramming genes (*OCT4, SOX2, KLF4, L-MYC, LIN28, dominant-negative p53)* into human PBMCs as described previously[1] and evaluated based on the expression of pluripotent stem cell markers and the elimination of transgenes. Three clones for each group were used for further analysis: control (201B7, 1201C1 and HKC1), MSA_A (A26, A31, A34), and MSA_B (B1, B2, B3). Data from the cell lines of patients are expressed as the average of the three clones. All experimental procedures for iPSC production were approved by Keio University School of Medicine Ethics committee (approval number, 20080016) and University of Tokyo Ethics committee (approval number, G2876-(2)).

Assessment of pluripotency of iPSCs by *in vitro* differentiation through embryoid body formation. iPSC colonies were detached from feeder layers and cultured in suspension as embryoid bodies (EBs) in non-treated culture dishes. After seven days of floating culture, the cells were transferred to Matrigel (CORNING)-coated culture plates to induce further differentiation. Differentiation was confirmed by immunocytochemistry for tridermic markers (endoderm: α-fetoprotein (AFP), mesoderm: α smooth muscle actin (αSMA), ectoderm: βIII-tubulin).

Teratoma Assay. Undifferentiated iPSCs (5×10^5 cells) were injected into the testes of 8-week-old male nonobese diabetic (NOD)/severe combined immunodeficiency (SCID) mice (Charles River Laboratories) as described previously[2]. Eight weeks after injection, the resultant tumors were dissected and fixed with 4% paraformaldehyde. Paraffin-embedded tissue sections were produced, and hematoxylin and eosin (H&E) staining was performed. Images were obtained using a BZ-9000 microscope (Keyence).

Immunocytochemical analysis. For immunocytochemical analysis, cells were fixed

with 4% paraformaldehyde for 15 min at room temperature and permeabilized with 0.2% Triton X-100 for 10 min. Cells were incubated overnight at 4℃ with primary antibodies diluted with blocking buffer (phosphate-buffered saline (PBS) containing 5% normal goat serum). After three washes with PBS, cells were incubated with Alexa 488-, Alexa 555-, or Alexa 647-conjugated secondary antibodies (Thermo Fisher Scientific) for 1–2 hrs at room temperature. Nuclei were stained with 10 µg/ml Hoechst 33258 (Sigma) or 0.5 µg/ml 4′,6-diamidino-2-phenylindole (DAPI) (Dojindo). After washing with PBS, the cells were examined using a confocal laser scanning microscope (LSM700) (Zeiss), Apotome (Zeiss) and BZ-9000 microscope (Keyence). The primary antibodies used in these analyses were as follows: NANOG (1:100; ReproCELL), OCT4 (1:500; Santa Cruz Biotechnology), βIII-tubulin (1:1,000; Sigma), cleaved-Caspase 3 (1:500; Cell Signaling Technology), AFP (1:250; RD Systems), αSMA (1:150; Sigma), MAP2 (1:1000; Merck Millipore), NeuN (1:500; Aves), VGLUT1 (1:500; Synaptic Systems), VGLUT2 (1:500; Abcam), VGAT (1:500; Frontier Institute), TH (1:500; Merck Millipore), and C-III core1 (1:200; Invitrogen).

Construction of plasmid vectors for gene editing. The CRISPR direct tool

(https://crispr.dbcls.jp/) was used for guide RNA design. For targeting exon 7 of the *COQ2* gene where two heterozygous mutations (p.R387* and p.V393A) are located on each allele, the following three kinds of guide RNAs were designed and corresponding pre-annealed oligo-DNAs were sub-cloned into pSpCas9n(BB)-2A-Puro (PX462)[3]: For the allele carrying p.R387*, sgCOQ2-L: CAGACCTGAGGATTGTTGGA and sgCOQ2(R387*)-R: ATTTATCTCCAAC**C**GAACAC; for the allele carrying p.V393A, sgCOQ2-L and sgCOQ2(WT)-R: ATTTATCTCCAAC**C**GAACAC. A targeting donor DNA plasmid (Fig. 2A) was constructed by modified Multisite Gateway-based method, as previously described[4,5]. Either the 5'- or 3'-arm was synthesized using the GeneArt Strings DNA Fragment (Thermo Fisher Scientific) with 18- to 21-bp homology extensions at both ends and inserted into the *Hpa*I site of the 5'-arm cloning vector (pENTR2-L3-HpaI-PBL-R1) or the 3'-arm cloning vector (pENTR2-R2-PBR-HpaI-L4), respectively, using a GeneArt Seamless PLUS Cloning and Assembly Kit (Thermo Fisher Scientific). Resultant 5'- and 3'-arm clones were confirmed by Sanger sequencing and assembled into a backbone vector (pUC-DEST-R3R4)[6] with a selection marker cassette clone (pENTR-L1-PGK-PurTK-L2)[4,5] using the Gateway LR Clonase II Enzyme mix (Thermo Fisher Scientific). The resultant clone, pUC-5'3'COQ2-PurTK (Fig. 2A), was further used as the targeting donor DNA vector. pDONR2 P3-P1R and pDONR2 P2R-P4

are derivatives of pDONR P3-P1R and pDONR P2R-P4[6], respectively, in which the *Hpa*I sites in the vector backbone were removed by mutagenesis.

CRISPR/Cas9 gene editing. Undifferentiated MSA_A26 iPSCs were dissociated into single cells using TrypLE Select, and were divided into 5 x 10^5 cells and centrifuged at 200 x g for 5 min. The supernatant was removed and the pellet was resuspended into 100 µl of Opti-MEM (Thermo Fisher Scientific) containing 10 µM Y27632, left and right Cas9-expressing guide RNAs and targeting donor plasmids.

Electroporation was performed using the NEPA21 electroporator (Nepa Gene). Beginning form the next day (D=1), cells were dispersed into StemFit AK02N containing 10 µM Y27632, every other day until colony-picking. To obtain cells with homologous recombination of PGK-PurTK into the *COQ2* locus, selection was performed twice with 1 µg/ml of puromycin for 24 hrs. Around D=13 to D=20, each single puromycin-resistant iPSC colony was picked up and plated on a well of iMatrix-511 coated 24-well plate. PCR genotyping for identification of knock-in clones was performed using the following primers: 5'COQ-PCR Fw and PGKP-Rv for the detection of the 5'-border of the knock-in allele and PuroR-Fw and 3'COQ2-PCR-Rv for the detection of the 3'-border of the

knock-in allele. PCR fragments from the knock-in clones were purified by PEG precipitation and confirmed for gene modification by Sanger sequencing using the following primers; Seq-COQ2-Fw and Seq-COQ2-Rv (Suppl. Table 1). Established knock-in iPSC clones with proper gene modification were maintained using the feeder-free culture method in 6-well plates.

The selection cassette was removed by introducing excision-only *piggyback* transposase (PBx)[7,8] into the established knock-in clones. Cells were transduced AdEFPBx, which is the adenovirus vector expressing PBx driven by EF1alpha promoter. (kindly provided by Dr. Yumi Kanegae, Jikei University School of Medicine) (D=0). Three days after infection (D=3), 10 µM ganciclovir (Sigma) was added to the media for selecting cells that were free from the PGK-PurTK cassette. After selections (D=22), each ganciclovir-resistant iPSC colony was picked up. PCR genotyping and Sanger sequencing of the PCR fragments were performed using the primers described above in order to confirm the correct excision of the cassette and the site-specific gene correction of the p.[R387*]/[V393A] mutations.

C-III core1 staining. The neurons derived by method 3 were fixed, boiled and stained

for βIII-tubulin and C-III core1, and counterstained with Hoechst. To quantify the IMM area of the neurons, the cytoplasmic area was extracted as shown in Supp. Figure S3A. The C-III core1-positive signals within the extracted area were then converted to monochrome. The IMM area was quantified from the digitized values using Image J software.

Sequencing Analysis. Genomic DNAs were isolated from each iPSC clone using KAPA MG Kit (KAPA BIOSYSTEMS). For sequencing of the mutations in the *COQ2* gene, all seven exons of *COQ2* were amplified by PCR. The PCR primers are listed in Suppl. Table 1. Strands of all amplicons were sequenced using a Big Dye terminator v1.1 cycle sequencing kit (Thermo Fisher Scientific) and an ABI Prism 3130xL Genetic Analyzer (Thermo Fisher Scientific).

Supp. Table S1. Primer list

Gene	Forward	Reverse	Assay
COQ2 Exon 1	TGAAGGAGGGCCACGAGAA	CCTAGAGTAAGCGACCACGATG	Sequencing
COQ2 Exon 2	GGGGTCCTTTGTGATTTGAG	TTCCATGCTGGATTTCTGTG	Sequencing
COQ2 Exon 3	TACCATGGGCCAGTCTCTTC	TGTGTGGTGAGTTACTTACACTTGC	Sequencing
COQ2 Exon 4	TTGTCTTAAAGTATTTCGTGGTTTC	ATCTCTCCATAAAAGTGTAGTTTGC	Sequencing
COQ2 Exon 5	CACTGAACACACTCCGATGC	TGCTTTCTCCTTAATTTGGTTC	Sequencing
COQ2 Exon 6	TCACCGCTTATGGTATATCTGC	TGCCAGGTAAACACAGAGGG	Sequencing
COQ2 Exon 7	TTTGCTGTTTTCTCCTCCG (Seq-COQ2-Fw)	AAATCTTCATCTTCAGGTTCTTAATTC (Seq-COQ2-Rv)	Sequencing
COQ2	GTGTATTTGCAGGGCTACCATGGGGAA (5’COQ-PCR Fw)	TGCTGTCCATCTGCACGAGACTAGTGAG (PGKP-Rv)	PCR genotyping
COQ2	CGAGCGGGTCACCGAGCTGCAAGAACTC (PuroR-Fw)	TGCTGCCCACACCCACATATACAAATGACT (3’COQ2-PCR-Rv)	PCR genotyping

COQ2	CACGGTGGTGACTTGCAG	TTCCAATGGGCTTGTCCAAC	qPCR
FOXG1	CCCGTCAATGACTTCGCAGA	GTCCCGTCGTAAAACTTGGC	qPCR
OTX2	ACAAGTGGCCAATTCACTCC	GAGGTGGACAAGGGATCTGA	qPCR
EN1	CCGCGCACCAGGAAGCTGAA	CAGCGCCAGGCCGTTCTTGA	qPCR
HOXB4	ACGTGAGCACGGTAAACCCCAA	ATTCCTTCTCCAGCTCCAAGACCT	qPCR
ACTB	TGAAGTGTGACGTGGACATC	GGAGGAGCAATGATCTTGAT	qPCR

qRT-PCR. Total RNAs were isolated using an RNeasy mini kit (QIAGEN), and cDNAs were prepared using an iScript^{TM} cDNA Synthesis Kit (BIO-RAD). qRT-PCR analysis was performed using SYBR *Premix Ex Taq* II (TAKARA BIO) (2X) /ROX Reference Dye II (50X) (TAKARA BIO) on a ViiA 7 real-time PCR system (Thermo Fisher Scientific). The value of each gene was normalized using beta Actin (ACTB) as an endogenous control. For the analysis of the expression of regional markers in neurospheres, neurospheres on day 12, which were generated by the neural induction controlling the regional identity (including method 2), and secondary neurospheres on day 14 after passage of method 3, were used. Data were presented as the expression relative to that in neurospheres cultured without any additives (untreated) on day 12. Reactions were carried out in duplicate, and data were analysed by using the comparative ($\Delta\Delta$Ct) method. The primer sets used in the experiments are listed in Suppl. Table 1.

LDH assay. The assay was performed with LDH Cytotoxicity Detection Kit (Takara) according to the manufacture's protocol. The enzyme reaction was performed under dark conditions at room temperature. The absorbance at wavelengths of 492 and 620 nm was measured using iMark microplate reader (BIO-RAD). The LDH activities were

determined by calculating absorbance difference.

High-Content Analysis. For the neural population assay, neurite length analysis, oxidative stress analysis and apoptosis analysis, stained plates and slides were imaged on the high-content cellular analysis system IN Cell Analyzer 6000 and set of 5 x 5 fields were collected from each well using the 20x objective lenses. Analysis (IN Cell Developer Toolbox v1.9.2) (GE Healthcare) began by identifying intact nuclei stained by Hoechst 33258, which were defined as traced nuclei. Each traced nucleus region was then cross-referenced with neuron marker (βIII-tubulin) to identify neurons. From these images, the ratio of βIII-tubulin-positive neurons was analysed. The CellROX-positive cell ratio and the cleaved-Caspase 3-positive cell ratio in βIII-tubulin-positive neurons was analysed using the traced images of each cell that have been described above. Using the above-described traced images of each cell, neurite length in βIII-tubulin-positive neurons were analyzed.

Statistical analysis. All experiments consisted of at least three independent replicates,

with biological or technical replicates indicated. All data are presented as means ± SE and were analyzed using paired *t*-test (two groups), one-way ANOVA followed by Turkey's post hoc test or Dunnett's test (more than two groups). Differences between means were considered statistically significant when $p < 0.05$. No statistical methods or power calculations were used to determine sample size; however these were kept constant between groups whenever possible.

Supplemental References

1. Okita, K. *et al.* An efficient nonviral method to generate integration-free human-induced pluripotent stem cells from cord blood and peripheral blood cells. *Stem Cells* **31,** 458–466 (2013).

2. Ohta, S. *et al.* Generation of human melanocytes from induced pluripotent stem cells. *PLoS One* **6,** (2011).

3. Ran, F. A. *et al.* Genome engineering using the CRISPR-Cas9 system. *Nat. Protoc.* **8,** 2281–2308 (2013).

4. Hosoya, M. *et al.* Cochlear Cell Modeling Using Disease-Specific iPSCs Unveils a Degenerative Phenotype and Suggests Treatments for Congenital Progressive Hearing Loss. *Cell Rep.* **18,** 68–81 (2017).

5. Ichiyanagi, N. *et al.* Establishment of in Vitro FUS-Associated Familial Amyotrophic Lateral Sclerosis Model Using Human Induced Pluripotent Stem Cells. *Stem Cell Reports* **6,** 496–510 (2016).

6. Sone, T. & Imamoto, F. *Methods for constructing clones for protein expression in mammalian cells*. *Methos Mol Biol* **801,** (2012).

7. Yusa, K., Rad, R., Takeda, J. & Bradley, A. Generation of transgene-free induced pluripotent mouse stem cells by the piggyBac transposon. *Nat. Methods* **6,** 363–

369 (2009).

8. Li, S.-H. *et al.* Mechanistic characterization and crystal structure of a small molecule inactivator bound to plasminogen activator inhibitor-1. *Proc. Natl. Acad. Sci. U. S. A.* **110,** E4941-9 (2013).
